# α‐ketoglutarate delays age‐related fertility decline in mammals

**DOI:** 10.1111/acel.13291

**Published:** 2021-01-15

**Authors:** Zhenzhen Zhang, Changjiu He, Yu Gao, Lu Zhang, Yukun Song, Tianqi Zhu, Kuanfeng Zhu, Dongying Lv, Jing Wang, Xiuzhi Tian, Teng Ma, Pengyun Ji, Wei Cui, Guoshi Liu

**Affiliations:** ^1^ National Engineering Laboratory for Animal Breeding Key Laboratory of Animal Genetics and Breeding of the Ministry of Agriculture Beijing Key Laboratory for Animal Genetic Improvement College of Animal Science and Technology China Agricultural University Beijing China; ^2^ Key Laboratory of Agricultural Animal Genetics, Breeding and Reproduction Education Ministry of China College of Animal Science and Technology Huazhong Agricultural University Wuhan China; ^3^ College of Animal Science and Technology Xinjiang Agricultural University Wulumuqi China; ^4^ Institute of Animal Science Chinese Academy of Agricultural Sciences Beijing China; ^5^ Department of Surgery & Cancer Imperial College London London United Kingdom

**Keywords:** mTOR, ovarian aging, reproduction, telomere, α‐KG

## Abstract

The fecundity reduction with aging is referred as the reproductive aging which comes earlier than that of chronological aging. Since humans have postponed their childbearing age, to prolong the reproductive age becomes urgent agenda for reproductive biologists. In the current study, we examined the potential associations of α‐ketoglutarate (α‐KG) and reproductive aging in mammals including mice, swine, and humans. There is a clear tendency of reduced α‐KG level with aging in the follicle fluids of human. To explore the mechanisms, mice were selected as the convenient animal model. It is observed that a long term of α‐KG administration preserves the ovarian function, the quality and quantity of oocytes as well as the telomere maintaining system in mice. α‐KG suppresses ATP synthase and alterations of the energy metabolism trigger the nutritional sensors to down‐regulate mTOR pathway. These events not only benefit the general aging process but also maintain ovarian function and delay the reproductive decline. Considering the safety of the α‐KG as a naturally occurring molecule in energy metabolism, its utility in reproduction of large mammals including humans deserves further investigation.

## Graphical abstract

Long term of α‐ketoglutarate (α‐KG) administration preserves the ovarian function, the quality and quantity of oocytes as well as telomere maintaining system in mice. α‐KG suppresses ATP synthase and alterations of the energy metabolism trigger the nutritional sensors to down‐regulate mTOR pathway. These events not only benefit the general aging process but also maintain ovarian function and delay the reproductive decline.

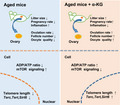

## 1 INTRODUCTION

Due to the postponing childbearing age in the modern humans, prolongation of the effective reproductive age becomes general requirement for females. Ovary plays a central role in mammalian reproduction. The ovarian reservation and oocyte competence decline with aging (Broekmans et al., 2009; Whitacre et al., 1998) and this is also referred as ovarian aging (synonym: reproductive aging) which often associates with fecundity reduction (Broekmans et al., 2009; Powers et al., 2006; Santoro et al., 2003). Ovarian aging occurs much earlier than general aging. The most obvious feature of ovarian aging is a significant decrease in the quality and quantity of oocytes. For example, a human female is born with approximately 400,000 primordial follicles with primary oocytes. These primary oocytes remain arrested in the prophase stage of meiotic division I, until sexual maturity. After beginning periods, an average of 1000 primary oocytes will be lost monthly and at middle age (around 37 years old), only around 25,000 eggs are left in a female. Thus, to preserve follicle pool and maintain oocyte quality will retard ovarian aging and extend female reproductive life.

The quality of oocytes usually depends on the health of post‐natal germ cells which are arrested for long period in mammals (in humans from age 10–50 years). During this arrested period, reactive oxygen species (ROS)‐related oxidative damage will be accumulated in these germ cells and it will negatively affect the quality and quantity of oocytes (Tarin, 1996). The mitochondrial DNA (mtDNA) of these germ cells is a easy target for ROS‐induced mtDNA mutations (Keefe et al., 1995) and telomere shortening (Huang et al., 2007; Lansdorp, 2005), which in turn impairs meiosis and embryonic development. The biological consequence is the reproductive failure and infertility. Thus, methods to reduce ROS level including caloric restriction (Chin et al., 2014) and antioxidant intervention, such as melatonin and resveratrol (Liu et al., 2013; Tamura et al., 2017), are widely used to delay ovarian aging.

In addition, to maintain the follicle pools preserving as more as possible of remaining primordial follicles is another strategy to delay the ovarian aging. A variety of genes and proteins can regulate follicle development, for example, GDF9, TGF‐β, Foxl2, and Sohlh1 activate of primordial follicle development, PTEN, Foxo3, AMH, Cyclin‐dependent kinase inhibitor p27, TSC, and mTOR inhibit primordial follicle activation. mTOR is the sensor of cellular nutrient, oxygen, and energy levels (Tokunaga et al., 2004), and it could modify the cellular metabolism. It has been reported that decreased TOR activity increases life span in *Saccharomyces. cerevisiae*, *Caenorhabditis. elegans*, and *Drosophila. melanogaster* (Harrison et al., 2009; Jia et al., 2004; Powers et al., 2006). The mTOR inhibitor, rapamycin, increases lifespan in mice (Fok et al., 2014; Harrison et al., 2009; Miller et al., 2014) and it may also be useful for treating/preventing several age‐associated conditions, including neurodegenerative diseases such as Alzheimer's disease and Parkinson's disease (Bove et al., 2011; Hasty, 2010). Various natural compounds, including epigallocatechin gallate, caffeine, curcumin, and resveratrol could inhibit mTOR in cell culture (Beevers et al., 2006; Bove et al., 2011).

α‐KG is a small molecule occurring in tricarboxylic acid cycle (TAC) and is ubiquitously present in organisms. In the cell, α‐KG is produced from isocitrate by oxidative decarboxylation catalyzed by isocitrate dehydrogenase. α‐KG can also be produced anaplerotically from glutamate by oxidative deamination using glutamate dehydrogenase, and as a product of pyridoxal phosphate‐dependent transamination reactions in which glutamate is a common amino donor. Finally, α‐KG is decarboxylated to succinyl‐CoA and CO2 by α‐KG dehydrogenase (encoded by *ogdh1*), a key control point in the TAC (Chin et al., 2014). mTOR signaling is involved in metabolism and activation of primordial follicle (Guo et al., 2018; Zhao et al., 2018), to identify mTOR signaling regulatory metabolites seems a feasible strategy fighting for reproductive aging. Metabolism and aging are intimately linked. Relatively low metabolic level is always associated with improvement of cellular functions and longevity in organisms (Bordone & Guarente, 2005). Chin et al first reports that α‐KG could prolong the lifespan of *C. elegans* by inhibiting mTOR and ATPase (Chin et al., 2014). However, there is still no research about the effect of α‐KG on reproductive aging.

Considering mTOR signaling is associated with follicle development and general aging, we hypothesize that ɑ‐KG is also a suitable candidate molecule to retard or even reverse the reproductive aging in mammals. In the current study, the effects of ɑ‐KG on reproductive aging are systemically investigated for the first time in mammals.

## 2 RESULTS

### 2.1 α‐KG exists in FF of mammals and decreases with age in human

**FIGURE 1:**
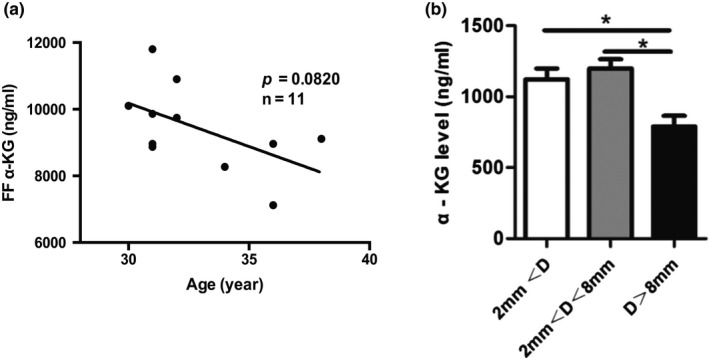
Evidence of α‐KG in FF of mammals. (a) The FF α‐KG levels and their association with age in human samples (*n* = 11). (b) The FF α‐KG levels in different sizes of porcine follicles. *D* indicated diameter of follicle (*n* = 15). Data are expressed as mean ±SEM. *p < 0.05

The potential correlations between follicle fluid (FF) α‐KG levels and age in human samples were examined. The FF α‐KG content was measurable in all samples with concentrations ranging from 7,120 to 11,800 ng/ml. As shown in Figure 1a, there was a tendency of the decreased FF α‐KG level with aging. Since α‐KG was found in the FF of human, we examine whether α‐KG was also presented in the FF of porcine. The results conformed that α‐KG is also detectable in FF of porcine. Its content ranges from 800 to 1,500 ng/ml, which is nearly 10 times lower than that found in the FF of human. It seems that α‐KG exists in FF of all mammals. Further studies indicated that the α‐KG concentrations were varied with the follicular development stages in porcine. The highest concentration was detected in the middle size (a diameter of 2–8 mm) follicles, followed by the small size (diameter <2 mm) and the large size (diameter >8 mm) (Figure 1b).

### 2.2 α‐KG promotes in vitro maturation of oocytes of porcine

**FIGURE 2:**
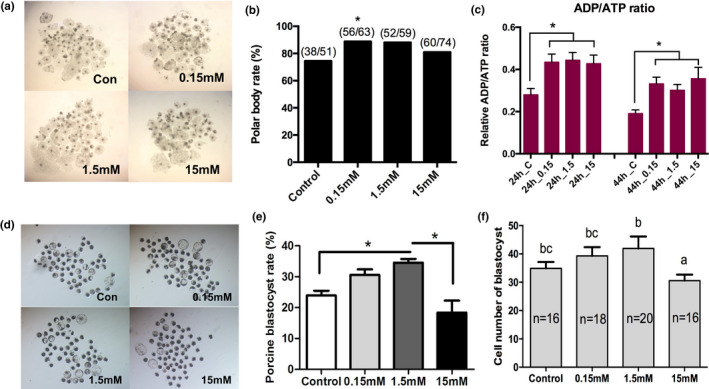
Effects of α‐KG on porcine oocytes in vitro maturation and embryo development. All porcine oocytes in vitro maturation cultured with different concentrations of α‐KG for 44 h. (a) Representative images of porcine oocytes in vitro maturation. (b) Average polar body rate of porcine oocytes. (c) Representative images of parthenogenetic activated porcine blastocysts (*n* = 15). (d) Average parthenogenetic activated porcine blastocyst rate. (e) Average cell number of parthenogenetic activated porcine blastocysts (*n* = 30). (f) ADP/ATP ratio in the oocyte in vitro maturation. The numbers in the x‐axis represent the time of culture and α‐KG concentrations. The data were expressed as Mean ±SEM. *p < 0.05. Letters on the panel represent *p* < 0.05 versus each other

Due to availability, porcine oocytes were selected to test the effects of α‐KG on it. The results showed that α‐KG treatment significantly promoted in vitro maturation of porcine oocytes and embryo development, indicated by the increased polar body excretion rate (Figure 2a,b) and the rate and total cell number of parthenogenetic blastocysts (Figure 2d‐f). The dose‐responsive studies identified the most effective dose of α‐KG in the current study was 1.5 mM. α‐KG treatment also significantly elevated the ratio of ADP/ATP during the maturation of porcine oocyte (Figure 2c) which confirmed the inhibited effect of α‐KG on the ATP synthase.

### 2.3 α‐KG delays fertility decline and pregnancy failure in advanced age mice

**FIGURE 3:**
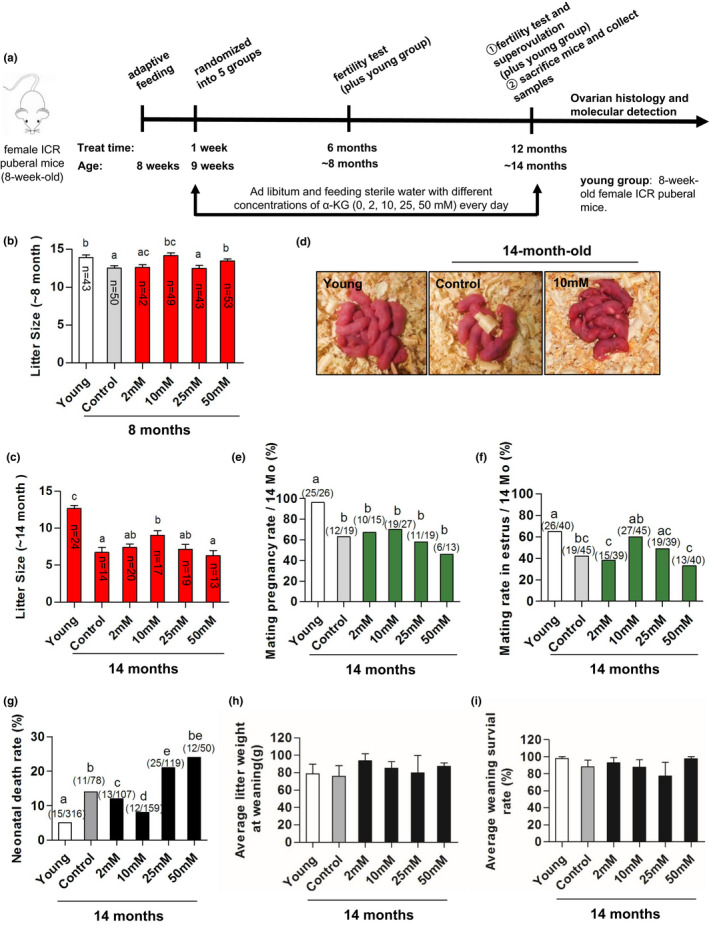
Fertility performances and offspring status among mice of different ages and treatments. (a) The sketch of experimental design. (b) Litter size of 8‐month‐old mice. The *n* above the bar chat indicated the number of successfully mated females (or the number of litters) and the total number of the mice for used in each group was about 50–55. (c) Litter size of 14‐month‐old mice. The *n* above the bar chat indicated the number of successfully mated females (or the number of litters) and the total number of mice for used in each group was about 40–45. (d) Representative photograph of offspring from mice at age of 8 weeks and 14 months old. (e) Average mating rate (with mating plugs). (f) Average pregnancy rate showing apparent large abdomen by Days 16–17 of mated mice in one estrus cycle based on the number of females that were successfully mated and plugged. (g) Neonatal death rate. (h) Average litter weight at weaning. (i) Weaning survival rate. The data were expressed as mean ±SEM (*N* = 25). 2 mM, 10 mM, 25 mM, and 50 mM: α‐KG concentrations in the drinking water. Control: mice without α‐KG treatment, Young: mice at age of 8 weeks. The letters represent *p* < 0.05 versus each other

It is not realistic to use large animals such as porcine to explore the mechanisms of α‐KG on reproductive aging. Therefore, the ICR female mice were selected to feed with different doses of α‐KG (2,10, 25, and 50 mM, respectively) from their 2 to 14 months old (Figure 3a). On the time of 6th or 12th month after α‐KG treatment, the fertility ability between the α‐KG treatment and the age‐matched control group was evaluated. In addition, 8‐week‐old mice group always served as young control (Figure 3a).

The litter size (pups born) was used to evaluate the fertility performance. From the dose‐responsive studies, it was identified that the most effective dose of α‐KG for the fertility performance was 10 mM. The results showed that the mice at the age of 8 months have already significantly reduced their litter size compared to 8‐week young mice (*p* < 0.05) (Figure 3b), while the litter size of 8‐month‐old mice treated with 10 mM α‐KG was not only significantly larger than that of their age‐matched control (*p* < 0.05) (Figure 3b) but also matched to the litter size of 8‐week‐old mice. At 14 months old, compared to 12–13 pups per litter for the 8‐week‐old young mice, the control mice only produced 6–7 pups per litter. However, 14‐month‐old mice treated with 10 mM α‐KG still produced 8–9 pups/litter which were significantly larger than that of their age‐matched controls (*p* < 0.05) (Figure 3c,d). It suggested that α‐KG especially in the concentration of 10 mM delayed the decline of reproductive ability.

In addition to litter size, mice at 14 months old had significantly lower mating and pregnancy rates than that of young group. This means that at 14 months old, some of female mice failed to pregnancy even when successfully mated (with the presence of mating plugs). When the mice were treated with α‐KG (2, 10, and 25 mM, respectively), they had significantly improved mating rate and pregnancy outcome at 14 months old compared to their age‐matched control (*p* < 0.05) (Figure 3e,f). These improvements seemed no direct association with estrus cycle since in this study α‐KG had limited impact on this cycle (Figure S1).

The neonatal death rate in mice of 14 months old was significantly higher than that of young mice, while the neonatal death rate in mice of 14 months old treated with 10 mM α‐KG was significantly reduced compared to their age‐matched control (*p* < 0.05) (Figure 3g). Besides, the average body weight and survival rate of offspring at weaning among different groups exhibited no significant differences (Figure 3h,i). Since α‐KG at 10 mM produced best result among other doses (Figure 3b,c), this dose was selected for the subsequent studies.

### 2.4 α‐KG increases the quality and quantity of oocytes in advanced age mice

**FIGURE 4:**
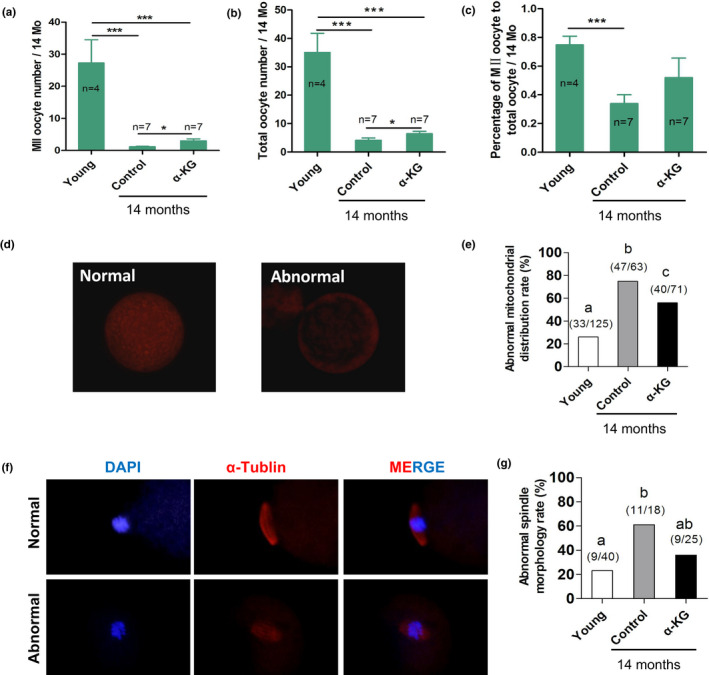
Effects of α‐KG treatment on quality and quantity of oocytes in mice with age. (a) Average number of MII oocytes. (b) Average number of total oocytes. (c) Rate of MII oocytes to total oocytes. (d) Representative images of mitochondrial distribution and (f) Spindle morphology of oocytes. (e) Statistical analysis of abnormal mitochondrial distribution and (g) abnormal morphology of spindle of oocytes. α‐KG: mice treated with 10 mM α‐KG in the drinking water for 12 months, Control: Age‐matched mice without α‐KG treatment, Young: Mice at 8 weeks old. Arrows: indicated the oocytes with normal mitochondria distribution. The number above the bar chart (e, g) indicated the number of sample, the denominator represents the total number of samples, and the numerator represents the number of differential samples. The data were expressed as mean ±SEM. *p < 0.05; ***p < 0.001. The letters in the panels: *p* < 0.05 versus each other

The quality and quantity of oocytes are important to the fertility of mammals. The main manifestation of reproductive aging is the decrease in both the quality and quantity of oocytes. In the study, the oocytes of young, control, and α‐KG (10 mM) groups were harvested by superovulation. These oocytes were calculated, classified according to their morphologies and subcellular structures including spindles and mitochondria. The results showed that the 14‐month‐old mice had remarkably fewer oocytes than that of 8‐week‐old mice (4.14 ± 0.80 vs. 35.00 ± 6.76) (*p* < 0.001) (Figure 4a). 14‐month‐old mice with α‐KG treatment had a significantly higher number of oocytes than that of their age‐matched controls (6.43 ± 0.87 vs. control 4.14 ± 0.80, *p* < 0.05) (Figure 4a). The percentage of oocytes with abnormal mitochondrial distribution (Figure 4d,e) and spindle (Figure 4f,g) were also significantly increased in 14‐month‐old mice compared to 8‐week‐old young mice (*p* < 0.05). Long‐term α‐KG treatment significantly reduced these abnormalities in 14‐month‐old mice (*p* < 0.05) (Figure 4d‐g). In addition, the relative expression of *Rec8* and *Smc1b* which related to cohesion was slightly higher but did not achieve significant in the ovaries of mice treated with α‐KG than that of their 14‐month‐old counterparts (Figure S2). The number of normal MII oocytes and the ratio of normal MII oocytes to total oocytes in 14‐month‐old mice were significantly lower than that of young mice (*p* < 0.05) (Figure 4b). α‐KG treatment slightly increased the ratio of normal MII oocytes to total oocytes but failed to reach statistical significance (Figure 4c) (*p* > 0.05).

### 2.5 α‐KG reduces the decline of primary and growing follicles in advanced age mice

**FIGURE 5:**
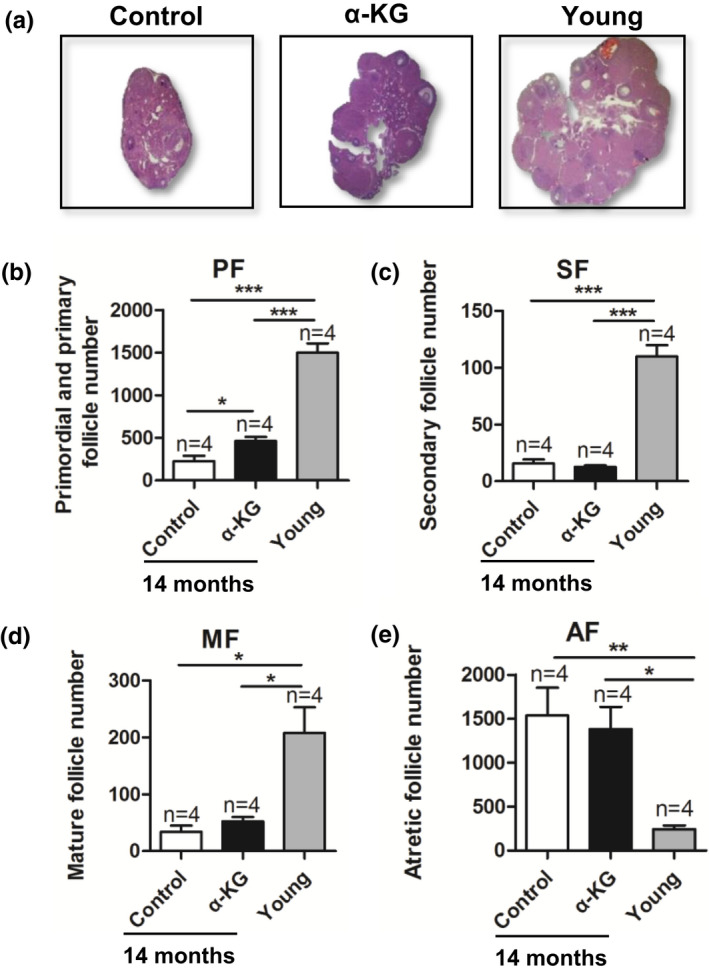
Effects of α‐KG on the ovarian morphology and follicle status of mice with age. (a) The representative photograph of ovarian morphologies. (b) The number of primordial and primary follicles. (c) Secondary follicles. (d) Matura follicles and (e) Atretic follicles. *n* = number of mice. α‐KG: mice treated with 10 mM α‐KG in the drinking water for 12 months, Control: age‐matched mice without α‐KG. Young: mice at 8 weeks old. The data were expressed as mean ±SEM. The *n* above the bar chat indicated the number of samples. *p < 0.05; **p < 0.01; ***p < 0.001

The ovaries of young (8 weeks old), 14‐month‐old control and 14 months old with 10 mM α‐KG treated mice were collected for continuously sectioning, and the numbers of different grade follicles were recorded. It is observed that in young mice there were large amounts of primary, primordial, growing and mature follicles located at the ovarian cortex and few numbers of atretic follicles and blood vessels localized to the medulla; in addition, the size of ovary in α‐KG group was larger than that in the control group (Figure 5a). In contrast, the mice at the age of 14 months had a remarkably less numbers of primordial and primary follicles, and few growing and mature follicles than that in the young mice (Figure 5a,b) (*p* < 0.01). Surprisingly, the number of primordial and primary follicles in mice of 14 months old treated with α‐KG (10 mM) was significantly higher than that of their age‐matched controls (Figure 5a,b) (*p* < 0.05). α‐KG treatment exhibited limited effects on the number of growing and mature follicles (Figure 5c,d), and this was also the case regarding the numbers of atretic follicles (Figure 5e). These data showed that long‐term (12 months) α‐KG treatment could significantly preserve primary and primordial follicle loss but not in other parameters tested in mice with age.

### 2.6 α‐KG reduces telomere shortening of advanced aged mice

**FIGURE 6:**
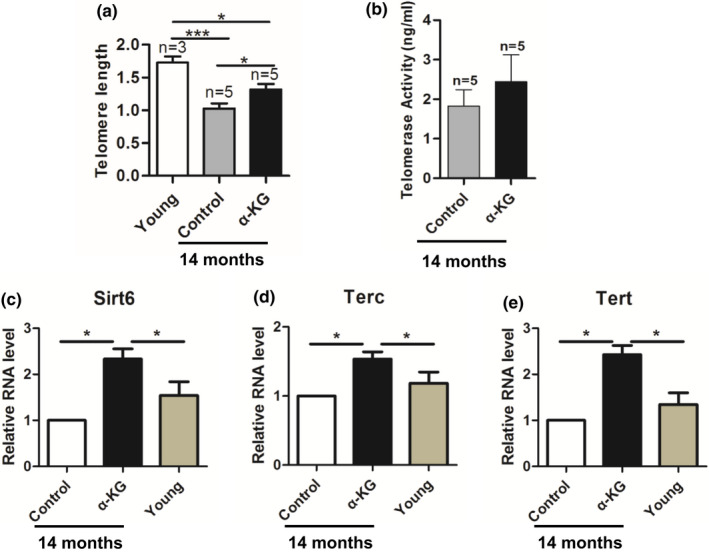
Effects of α‐KG on ovary telomere shorting rate and expression of telomerase‐related genes in ovarian tissue of mice with age. (a) Relative ovary telomere length shown as the T/S ratio by quantitative real‐time PCR (q‐RT‐PCR) analysis. (b) Telomerase activity of ovaries by enzyme‐linked immunosorbent assay analysis. (c–e) Relative gene expression of *Tert*, *Terc*, and *Sirt6* in the ovaries analyzed by quantitative real‐time PCR. α‐KG: mice treated with 10 mM α‐KG in the drinking water for 12 months, Control: age‐matched mice without α‐KG, Young: mice of 8 weeks old. The data were expressed as mean ±SEM (*n* = 5). *p < 0.05; ***p < 0.001.

The results showed that the telomere length was significantly shorter in the ovaries of 14‐month‐old mice than that in the 8‐week‐old young mice (*p* < 0.05) (Figure 6a). However, the telomere length in 14‐month‐old mice treated with α‐KG was significantly longer than that in their age‐matched control mice (*p* < 0.05) (Figure 6a). Consistently, the telomerase activity in 14‐month‐old mice treated with α‐KG was also slightly higher than that in their age‐matched controls but it did not achieve significant difference (*p* > 0.05) (Figure 6b). *Terc*, *Tert*, and *Sirt6* play an important role in telomere length. The results showed that the relative expression of *Sirt6*, *Tert*, and *Terc* was significantly down‐regulated in the ovaries of 14‐month‐old females compared to young ones, while these reductions were significantly recovered with α‐KG administration compared to their age‐matched mice (*p* < 0.05) (Figure 6c‐e). Compared with the control group, the anti‐oxidation SOD enzyme activity of the ovaries in the α‐KG group increased slightly at 14 months of age (Figure S2c), but did not reach significant. These results indicated that long‐term application of α‐KG could slow down the shortening of telomeres, thereby delaying reproductive aging.

### 2.7 α‐KG inhibits the mTOR signaling in mice

**FIGURE 7:**
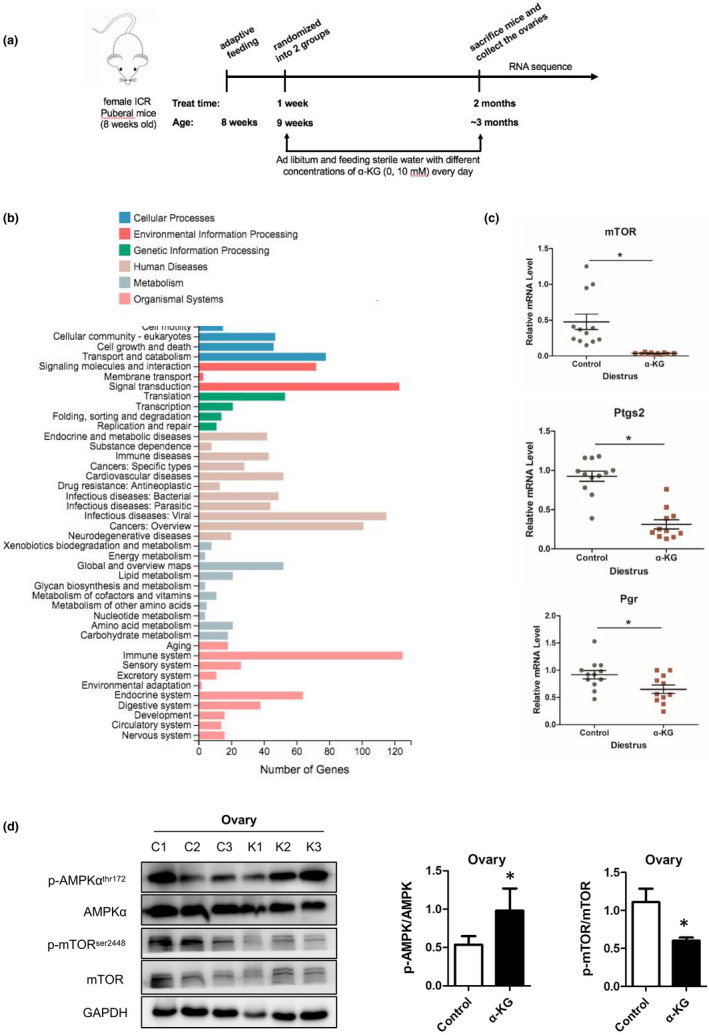
Effects of α‐KG on the AMPK/mTOR pathway in granulosa cells and ovary of mice. (a) The sketch of experimental design. (b) The KEGG pathway regulated by α‐KG. (c) Relative gene expression levels of *m‐TOR*, *Pgr*, and *Ptgs2*, respectively. (d) The representative images of expression of AMPK, p‐AMPK (thr172), mTOR, and p‐mTOR (Ser2448) in ovary of mouse following administration of α‐KG for 2 months as well as their statistical analyses.*p < 0.05

In order to study the specific molecular mechanism of α‐KG on the ovary, RNA‐seq was used to screen the key pathways which are related to aging (Figure 7a). The KEGG pathway results showed that α‐KG had profound associations with aging, immune system, immune diseases, infectious diseases, the cell growth and death, replication and repair, digestive system, folding, sorting and degradation et al (Figure 7b). And α‐KG targeted mTOR signaling (Figure S5)，q‐RT‐PCR (Figure 7c) and Western blotting (Figure 7d) results confirmed that the mTOR was significantly down‐regulated by α‐KG. Besides, the AMPK was significantly up‐regulated in α‐KG group. Similar results were found in kidney and spleen of 14‐month‐old mice fed with α‐KG treatment and *in vitro* culture mice granulosa cell with α‐KG treatment (Figure S3a,b).

Based on the above data, we further look at the RNA‐seq data and see whether genes involved in mitochondria, inflammation or immune response and telomere/telomerase‐related genes are regulated by α‐KG. The RNA‐seq data showed that 17 mitochondrial‐related genes were significantly changed by α‐KG (Table S2). The up‐regulated genes including *Slc25a32*, *Mftc*, *Slc25a24*, *Scamc1*, *Mrps35*, *Cyp11b1*, *Vars2*, *Kiaa1885*, and *Vars2l*, which are related to the mitochondrial folate transporter/carrier, Calcium‐binding mitochondrial carrier protein SCaMC‐1, 28S ribosomal protein S35, steroid 11‐beta‐monooxygenase, mitochondrial, long‐chain specific acyl‐CoA dehydrogenase, mitochondrial et al. The down‐regulated genes including *Mfn2*, *Rpl35a*, *Acsm3*, *Cox6a2*, *Hspb7*, *Kmo*, *Acaa1b*, and *Lyrm7*, which are related to transmembrane GTPase MFN2, 60S ribosomal protein L35a, Acyl‐CoA synthetase medium‐chain family member 3, cytochrome c oxidase subunit 6A2, Cytochrome c oxidase polypeptide VIa‐heart, heat shock protein beta‐7 (HspB7), kynurenine 3‐hydroxylase, 3‐ketoacyl‐CoA thiolase B, Acetyl‐CoA acyltransferase B, complex III assembly factor LYRM7 et al.

The immune response‐related pathway was significantly modified by α‐KG. The RNA‐seq result showed that the expressions of 59 genes were significantly altered by α‐KG (Table S2). The up‐regulated genes including *H2‐Ea*, *H2‐Ea‐ps*, *Klra16*, *Ly‐49P*, *Ly49P*, *H2‐Q4*, *H2‐GS10*, *H2‐Gs10*, *H2‐gs10*, *H2‐Q1*, and *Muc5b*, which are related to MHC class II antigen IEk‐alpha, natural killer cell receptor Ly‐49P1, MHC class I like protein GS10, Mucin 5, subtype B, and tracheobronchial. The down‐regulated genes including *Mapk14*, *Nlrp12*, and *Cfd*, which are related to mitogen‐activated protein kinase 14, NACHT, LRR and PYD domains‐containing protein 12, Adipsin et al. The telomere maintenance‐related genes in the ovary of 2‐month‐old mice treated with 10 mM α‐KG for 2 months have no significant change.

## 3 DISCUSSION

α‐KG is a key intermediate in the Krebs cycle, coming after isocitrate and before succinyl‐CoA. Herein, for the first time, α‐KG is found present in human FF and its level decreased with age. In porcine, the concentration of α‐KG of apoptotic follicles was significantly lower than healthy follicles. Moreover, α‐KG supplementation increased the in vitro maturation of porcine oocytes. In mice, long term of α‐KG supplementation in drinking water retards the fertility decline during aging as indicated by increased litter size and pregnancy rate after its treatment. In addition, α‐KG increases the number of follicle and preserves telomere length, as well as the quantity and quality of oocyte. These results strongly suggest that α‐KG retards the fertility decline with aging in mammals.

The reproductive aging appears earlier than general aging in mammals. For example, mice at the 8 months of age which is consider young have already shown the reduced reproductive capacity and at age of 14 months (equivalent to human 30–40 years old) their reproductive capacity has declined to half of the young females. This decline is retarded by α‐KG feeding. 10 mM α‐KG in drinking water is the most effective dose tested and lower and higher than this dose has reduced effects. These may be caused that α‐KG inhibits the electron transport chain (ETC) of mitochondria and too high or too low will lead to insufficient or overshooting inhibition of the ETC. This is in consistent with the previous observation by us and others, in *Caenorhabditis. elegans* α‐KG at 8 mM concentration retard aging while other concentrations have limited effects (Chin et al., 2014), and in mice, 150 μM ɑ‐KG was the best concentration of promoting the development of early‐stage embryos by reducing ATP and increasing the demethylation level (Zhang et al., 2019).

We also found α‐KG supplementation increased the ADP/ATP ratio of oocytes and modify mitochondria‐related gene expression in RNA‐seq. The results confirmed that mitochondria are a primary target of α‐KG. Besides, α‐KG is a small molecule, and it has the capacity to pass through the cell membrane. Aussel et al have observed that α‐KG enters inside of fibroblasts with easy by simple diffusion (Aussel et al., 1996). Other report suggests a relatively weak transmembrane activity of α‐KG but it can be increased by the use of its esters (Koivunen et al., 2007; MacKenzie et al., 2007). These differences may be cell specific. Judging from our results, α‐KG can be easily delivered into oocytes as it does in fibroblasts mentioned above.

In mammals, the females are born with a fixed number of oocytes, and these oocytes will lose during aging until few or none. Thus, reproductive aging is generally characterized by gradual decline in oocyte quality and quantity.Aneuploidy, DNA oxidative damage, meiotic spindle impairments, or mitochondrial aggregation occurred in oocytes of mammals with increased age. In the study, at 14 months of age, the quality (oocytes with abnormal spindles and mitochondria) and quantity (good MII and total oocytes) of oocytes significantly reduced. These aging‐related ovarian changes lead to reproductive aging in advance of the general aging and the decline of reproductive functions. α‐KG reduced these abnormalities. *Smc1b* and *Rec8* genes are related to chromatid cohesion and DNA recombination during meiosis (Kurahashi et al., 2012; Tsutsumi et al., 2014). Both of them are slightly up‐regulated in the ovaries of 14‐month‐old α‐KG treated group, but failed to achieve significant, which is in line with the better spindle and greater litter size. Besides, the number of primordial and primary follicles of 14‐month‐old mice treated with α‐KG (10 mM) was significantly higher than that of their age‐matched control group. These results confirmed that α‐KG delays the decreases of oocyte quality and quantity with aging.

Telomere is a life timer of cell and gradually shorted with aging. Telomere dysfunction may cause meiotic defects related to reproductive aging, miscarriage, and infertility. Telomerase deficiency and shorter telomeres contribute to the age‐related decline in ovarian follicle number and infertility (Broekmans et al., 2009). In the present study, we found ɑ‐KG significantly reduced the telomere short of the ovaries in mice at 14 months of age compared to their age‐matched untreated control, and the telomerase activity of ɑ‐KG group was slightly enhanced but failed to achieve significant. Moreover, the expressions of telomerase reverse transcriptase (*Tert*), telomerase RNA component (*Terc*), and sirtuin 6 (*Sirt6*) in ovaries of 14‐month‐old α‐KG group were all significant up‐regulated. Tert is the catalytic subunit of telomerase, and it forms the most important unit of the telomerase complex together with Terc. And Sirt6 plays a role in the maintenance of telomeres (Tennen et al., 2011) and also participates in other aging‐related processes (Frye, 2000), including DNA repair, sugar fermentation, and inflammation. However, there was no significant change in telomere maintenance‐related genes in the ovaries of 2‐month‐old mice treated with 10 mM α‐KG, which is predictable because the ovaries of young animals are at their peak of function; thus, it had limited room for improving.

Inflammation and immune response are also closely related to aging. The level of IL‐6 was significantly lower in the ovaries of mice treated with α‐KG than that of their 14‐month‐old counterparts (Figure S2d). The immune response‐related genes were significantly modified by α‐KG. This was consistent with Liu et al. (2018) that α‐KG has function in inflammation regulation.

In consistent with previous reports (Chin et al., 2014), our RNA‐seq data revealed that mTOR plays an important role in delaying fertility decline in α‐KG group. Both the gene and protein level of mTOR in ovary (Figure 7d), spleen, and kidney (Figure S3a) of α‐KG treated mice and in vitro cultured granulose cells with α‐KG were significantly down‐regulated. Previous reports have proved that *mTOR* plays significant roles in follicle development (Goldman et al., 2017; Guo et al., 2018; Zhao et al., 2018). As little as a twofold to fourfold reduction in mTOR activity would preserve ovarian function and normal birth numbers (Goldman et al., 2017). In our study, the relative level of p‐mTOR/mTOR ratio in ovaries of 10 mM α‐KG treated group was about one third of the control, which is beneficial to preserve ovarian function. Despite follicle development regulation, mTOR is the main nutrient‐sensing protein linked to the regulation of aging and life span (Zhao et al., 2018). The expression level of up‐stream protein AMPK was also detected and analyzed after α‐KG treatment. As a cellular energy receptor which responds to low levels of ATP (Ke et al., 2018), AMPK pathway has also been significantly up‐regulated with α‐KG treatment observed in our study. These results together showed that α‐KG might delay follicle activation and senescence in mice via up‐regulating the phosphorylation of energy‐sensing protein AMPK and down‐regulating the phosphorylation of mTOR (Chin et al., 2014; Goldman et al., 2017).

Interestingly, we found that α‐KG enhanced both the food intake and weight both in mice and piglets (Figure S4a,b). These were in line with previous studies, ɑ‐KG as promotes appetite and growth from the early life to late stages of life in human (Tian et al., 2020). The ornithine ɑ‐ketoglutarate (OKG) could significantly increase the concentration of insulin growth factor and glutamine and the growth slope of height in growth retarded children receiving long‐term total parentaral nutrition (Moukarzel et al., 1994). After stopping taking OKG, the promoting effects were significantly reduced. In the elderly (Blonde‐Cynober et al., 2003), OKG improves clinical outcome in chronic malnutrition by increasing appetite and body weight gain and improving healing. However, in *Caenorhabditis. elegans*, animals did not view α‐KG treated food as less or more favorable, and there was no significant change in food intake, pharyngeal pumping, foraging behavior, body size, or brood size in the presence of α‐KG (Chin et al., 2014). This may be explained by that the human leptin has no apparent *C. elegans* orthologue (Hashmi et al., 2013) so that the inhibited TOR could not affect the appetite of *C. elegans*. In mammals, inhibition of mTOR signaling could blunts leptin's anorectic effect so that increases food intake and body weight (Cota et al., 2006).

α‐KG has a good solubility and is relatively stable in water solutions (McLain et al., 2011). And α‐KG could be fully metabolized by the body and no excretion of the compound in the pure form with urine or faces is observed. Sodium salt of 14C‐labeled α‐KG revealed the presence of α‐KG carbon in several tissues (liver, brain, skin, muscle, and bone tissue) already after 3 h of administration of the compound (Filip & Pierzynowski, 2008; Hashmi et al., 2013). Ingested α‐KG was absorbed in the upper small intestine and then metabolized in enterocytes in animal. During the first pass metabolism in the intestinal mucosa, about 40% of α‐KG is degraded to CO_2_ (Junghans et al., 2006). The remaining part of α‐KG may be used in various anabolic pathways, both in the enterocytes and in peripheral tissues (Filip & Pierzynowski, 2008). So, in the current study, α‐KG in water is stable and could be fully used by animal.

**FIGURE 8:**
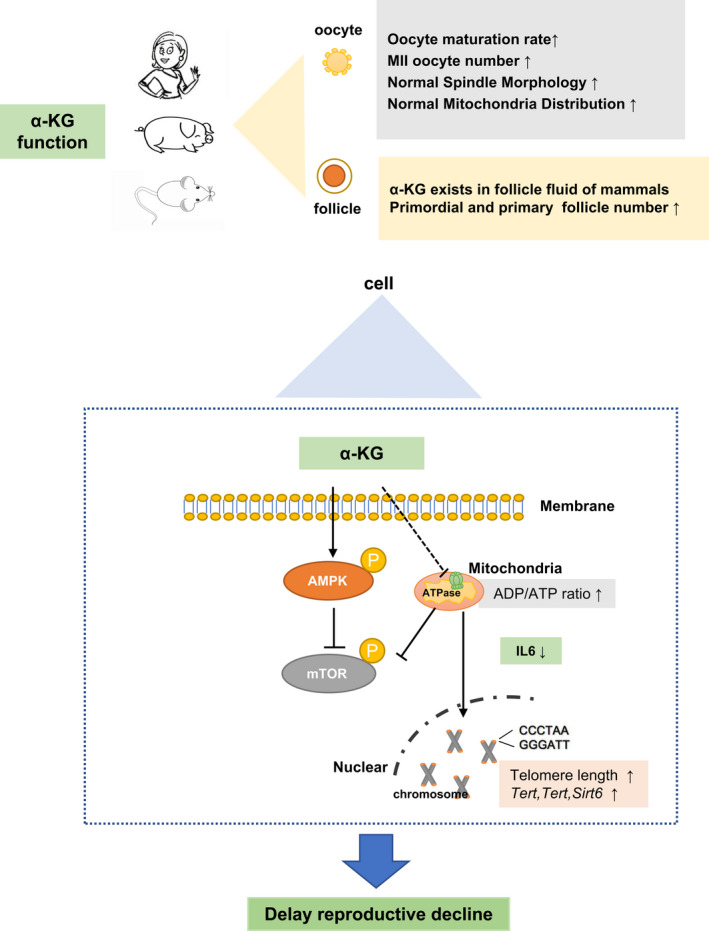
Physiological and molecular effects of α‐KG

In conclusion, the present study demonstrates the importance of ɑ‐KG on reproductive aging in mammals including mice, pigs, and humans. The molecular mechanisms are mainly attributed to that ɑ‐KG improves the telomere maintenance system, down‐regulates mTOR nutritional sensing signaling pathway, and up‐regulates the energy metabolism sensing signaling pathway of AMPK. All of these may be resulted from that ɑ‐KG mildly suppresses the ATPase to balance the ATP production. The altered energy metabolism triggers a serial downstream signaling pathway. These are summarized in Figure 8.

Considering ɑ‐KG being a natural metabolite of TCA cycle, these findings warrant further research regarding the utility of ɑ‐KG on reproductive aging in large mammals including humans.

## 4 MATERIALS AND METHODS

Pregnant mare serum gonadotropin (PMSG) and human chorionic gonadotropin (hCG) were purchased from Ningbo Hormone Products Co., Ltd. Alpha‐ketoglutarate and other reagents, unless specified, were purchased from Sigma‐Aldrich Chemical Co.

### 4.1 Mice feeding and mating

Eight‐week‐old female ICR mice were purchased from Vital River Laboratory Animal Technology Co. Ltd. The animals were housed in the no‐pathogen animal facility of the Experimental Animal Center of China Agricultural University with 12 h:12 h light: dark cycle, at temperature 22–25°C. Mice were randomized into 5 groups: One group was served as a control without treatment, and the other four groups were supplemented with α‐ketoglutaric acid (Sigma Chemical Co.) at different concentrations of 2, 10, 25, and 50 mM, respectively, in the drinking water). The stock solution of α‐ketoglutaric acid was stored at a temperature of 4°C. Sterile drinking water was freshly prepared using α‐KG powder, and drinking water was changed every 3 days. According to the concentration of α‐KG in each group, the amount of water intake and body weight, the daily intake of α‐KG per mouse is calculated as mg/body weight: 2 mM, 0.55 mg/kg body weight; 10 mM, 3.17 mg/kg body weight; 25 mM, 8.71 mg/kg body weight; 50 mM, 18.00 mg/kg body weight. After 6–12 months of treatment, some mice were randomly selected for reproduction. The litter sizes were recorded for assessing of fertility. Others were used to assess follicle reservation, oocyte number and quality, telomere length and telomerase activity, expression of genes associated with senescence and DNA damage. Mice at the age of 8 weeks were served as young controls.

Eight‐week‐old male mice with proven fertility were used for breeding experiments. In later afternoon, a female and a male were placed in the same cage for mating. In next morning, the females with the plugs were separated from the male and placed singly in the cage, and the females without mating plugs were allowed to mate for another night. Females who did not have a plug for three consecutive days were not used for breeding experiments. Post‐algebras were recorded within 1 day after delivery.

### 4.2 Measurement of food & water intake and body weight

#### 4.2.1 Mice

The body weight was monitored weekly, and the food and water intakes were recorded every 3 day.

#### 4.2.2 Piglet

The same batch of weaned piglets were selected and divided into two groups. Their weaning body weight and food intake were recorded. α‐KG was mixed into the food and made its available approximately 0.3 g/kg/day. After 10 days of continuous feeding, the body weight was recorded again and, then, calculate the average body weight gain.

### 4.3 Recording the estrus cycle using vaginal smear method

The estrus cycle was identified by vaginal smear. First, the changes in the vulva and the characteristics were observed and recorded. A small amount of physiological saline was dropped into the vagina of the mouse with a pipette and mixed well with the secreted products of the vagina by repeated pi‐petting. The mixture was aspirated and smeared on a glass slide. After drying, hematoxylin–eosin staining was performed, followed by punching, drying, and microscopic examination.

### 4.4 Follicle counting

Ovaries were randomly collected from different groups, and follicle counting was performed as described previously (Liu et al., 2013).

### 4.5 Oocyte collection

Female mice from the different groups (8‐week‐old, 8‐month‐old, and 14‐month‐old mice with/out 10 mM α‐KG) were super‐ovulated by injection of 5 IU PMSG, 46–48 h later followed by injection of 5 IU hCG. Females were sacrificed, and oocytes enclosed in cumulus masses were collected. Cumulus cells were removed by pi‐petting after brief incubation in 0.01% hyaluronidase prepared in 0.1%‐PVA‐PBS.

### 4.6 Oocytes mitochondrial distribution and spindle morphology assay

Oocytes mitochondrial distribution and spindle morphology assay were performed as previous report (He et al., 2016).

### 4.7 Measurement of telomere length and telomerase activity

Telomere length and telomerase activity were measured as previous report (Liu et al., 2013).

### 4.8 SOD activity assay in ovary

14‐month‐old female mice were killed by cervical dislocation, and ovaries were collected for SOD detection. The level of SOD was detected with the SOD assay kit (A001, Nanjing Jiancheng Bioengineering Institute) followed the manufacturer's instructions.

### 4.9 IL‐6 assay

Blood was collected from caudal vein of mice. After 30 min clotting, the serum was obtained by centrifugation (1500 *g*) at 4°C for 10 min. The level of IL‐6 was detected by Mouse IL‐6 ELISA Kit (ab100712; Abcam) followed the manufacturer's instructions.

### 4.10 Isolation and culture of murine granulose cells

Three‐week‐old female mice were super‐ovulated by consecutive injection of PMSG and hCG. The mice were sacrificed, and the ovary was collected; then, the follicles were poked to release the granulosa cell with 1 ml injector under inverted microscope. The samples were transferred into 15‐ml tube containing 1% FBS‐DMEM/F12 and centrifuged for 5 min (140 g), the medium was discarded, and the granulosa cells were washed with PBS. The above process was repeated for three times. The granulosa cells were placed into 12‐well plate and cultured with 10% FBS‐DMEM/F12 in 5% CO2 incubator for 2 h, and then, the granulosa cells were washed with PBS to isolate COCs and dead cells. Then, granulosa cells were cultured again in 10% FBS‐DMEM/F12, and medium was changed every 24 h. When the cell density achieves 80% confluence, different concentrations of α‐KG were added to the medium incubated for 4 h.

### 4.11 Real‐Time PCR

Total RNA was extracted using Trizol (Invitrogen Inc) and immediately reverse transcribed using PrimeScript^™^ RT reagent Kit with cDNA Eraser (TaKaRa Bio Inc.). Qualitative polymerase chain reaction (qPCR) amplification was performed by LightCycler 480 SYBR Green I Master Mix (Roche Applied Science) on LightCycler 480 II PCR machine (Roche Applied Science). At least three parallel samples were analyzed for each gene. The PCR system was consisted of 10 μl SYBR Green, 1 μl 10 μM forward and 1 μl 10 μM reverse primers, 2 μl template, and 6 μl ddH_2_O. The procedure was as follows: 95°C for 10 min; 35 cycles of 95°C for 10 s and 60–62°C for 8–15 s; melting curve from 65 to 95°C, increasing in increments of 0.5°C every 5 s. Normalization was performed using the housekeeping gene *actin* as a control. Primer sequences are listed in Table S1. Relative mRNA expression was calculated by the 2^−△△ct^ method.

### 4.12 Western blotting

Granulosa cells, ovary, kidney, and spleen were washed three times with 0.1% PVA‐DPBS and lysed, respectively, in sample buffer (Bio‐Rad Lab.) containing 62.5 mM Tris–HCl (pH6.7), 5% 2‐mercaptoethanol, 2% sodium dodecyl sulfate, 10% glycerol, and 0.002% bromophenol blue, then denatured by heating to 100°C for 5 min and frozen at −80°C until use. The proteins were subjected to SDS‐PAGE using a 10% polyacrylamide gel and then transferred to a polyvinylidene fluoride membrane (Millipore, 0.45‐lm pore size) for 2.5 h under a 300‐mA electric current. After blocking the nonspecific binding sites by overnight incubation in Tris‐buffered saline (25 mM Tris and 150 mM NaCl, pH 7.6) containing 5% nonfat milk and 0.2% Tween 20, the membranes were incubated with the primary antibody for 2 h at 37°C. The primary antibody used was anti‐mTOR (2983s, Cell Signaling, 1:1000), phospho‐mTOR (5536s, Cell Signaling, 1:1000), AMPK (2532s, Cell Signaling, 1:1000), and phospho‐AMPK (2535s, Cell Signaling, 1:1000). The membranes were stripped with a buffer containing 100 mM 2‐mercaptoethanol, 2% SDS, and 62.5 mM Tris–HCl (pH 6.7), then re‐probed with a mouse monoclonal antibody directed against GAPDH (10494‐1‐AP; Proteintech, 1:5000) to confirm equivalent protein loading. The bound antibodies were detected by incubation with a secondary antibody (Goat anti‐rabbit IgG‐HRP, SA00001‐2, Proteintech, 1:10,000; Goat anti‐mouse IgG‐HRP, SA00001‐1, Proteintech, 1:10,000). The images were scanned in an Image Quant LAS 4000 mini luminescent image analyzer (GE Healthcare Bio‐Sciences). The protein levels were evaluated by densitometry using Quantiy One software (v. 4.52; Bio‐Rad Lab).

### 4.13 High‐Throughput RNA sequencing and biological analysis

The total RNA extracted from the mouse ovary was lysed by TriZol method. The extracted total RNA was stored in RNase‐free water for the synthesis of first‐strand cDNA. Then, the first‐strand cDNA was amplified by the full‐length LD‐PCR, and the double‐chain cDNA (ds cDNA) was purified by AMPure XP beads and was quantified by using Qubit. The ds cDNA was interrupted by an ultrasound using the Covaris system, the interrupted two‐strand short fragment was repaired at the end, the A tail and joint were added to the sequencing connector, and then, the AMPure XP beads were used for purification. Fragments (~200 bp) were selected and enriched by PCR for the generation of a cDNA library. After the completion of the library construction, first using Qubit2.0 for the initial quantification and to dilute the library to 2 ng/µl and then using AGILENT2100 to detect the library insert size. When the insert size was consistent with the expected size, the effective concentration of the library was accurately quantified by using the Q‐PCR method (effective concentration of library >2 nM) to ensure the quality of the library. After the inspection of the library, according to the requirements of effective concentration and target data volume, different libraries were utilized for HiSeq sequencing. The sequenced reads were mapped to the reference mouse genome (mm10). The gene expression intensity was normalized as the fragments per kilo‐base of exon per million fragments mapped (FPKM). The gene ontology (GO; http://www.geneontology.org) was used to annotate biological themes, and The Kyoto Encyclopedia of Genes and Genomes (KEGG; http://www.genome.jp/kegg/) was used to find the associated pathways. Novogene (Beijing, China) has assisted to complete the sequencing and the data annotation.

The input data of the gene differential expression analysis are the readcount data that are obtained for analysis of the gene expression level. The analysis is mainly divided into three parts: (a) to normalize the read count; (b) to calculate the hypothetic probability (*p* value) according to the model (DESeq); and (c) to perform a multiple hypothetic test to obtain the FDR value (error discovery rate). DESeq software was used to analyze the differential expression of genes for different situations. To increase the power to detect biologically meaningful functions, a relative relaxed criterion of fold change (twofold) and a *p* < 0.05 were used to filter differential expressed genes for biological analysis.

### 4.14 Statistical analysis

Data are expressed as mean ±S.E.M. Statistical analyses were performed using the univariate analysis of chi‐square test or variance (ANOVA) followed by the Student *t* test with the aid of SPSS 20.0 statistical software, *p* < 0.05 was considered significant, and *p* < 0.01 was considered highly significant.

## ETHICS APPROVAL

### Animals

The studies are strictly following the protocol of the Animal Welfare Committee of China Agricultural University (permission number: CAU20150915‐1).

### Human subjects

The human study and all experimental procedures were approved by the Ethics Committee of Hebei Zhuozhou Hospital according to the Council for International Organizations of Medical Sciences. The protocol number is ZZSYY/YXLL‐2019‐02. We recruited 11 individuals of Chinese ancestry with age from 30 to 39 years old. All subjects were recruited from the Reproductive Medical Center at Hebei Zhuozhou Hospital. Written informed consent was obtained from all participants. The human follicle fluid was collected from volunteers after egg retrieval.

## CONFLICT OF INTEREST

The authors declare that there is no conflict of interest that could be perceived as prejudicing the impartiality of the research reported.

## AUTHOR CONTRIBUTIONS

Zhenzhen Zhang, Changjiu He, and Guoshi Liu designed the experiments and performed data analysis. Zhenzhen Zhang, Changjiu He, Yu Gao, Yukun Song, Tianqi Zhu, Dongying Lv, Jing Wang, Xiuzhi Tian, Teng Ma, and Pengyun Ji carried out the experiments. Zhenzhen Zhang, Changjiu He, and Guoshi Liu wrote the manuscript. Guoshi Liu, Wei Cui, and Lu Zhang revised the paper and contributed to critical discussions. Guoshi Liu supervised the research group. All the authors read and approved the final manuscript.

## Supporting information

Figure S1

Figure S2

Figure S3

Figure S4

Figure S5

Tables S1–S2

## Data Availability

The data used to support the findings of this study are available from the corresponding author upon request.
