# Supplementary figures and images for: α‐ketoglutarate delays age‐related fertility decline in mammals
Source: Aging Cell. 2021 Jan 15;20(2):e13291. doi: 10.1111/acel.13291 (PMC7884030; doi:10.1111/acel.13291)

S\_Figure 1

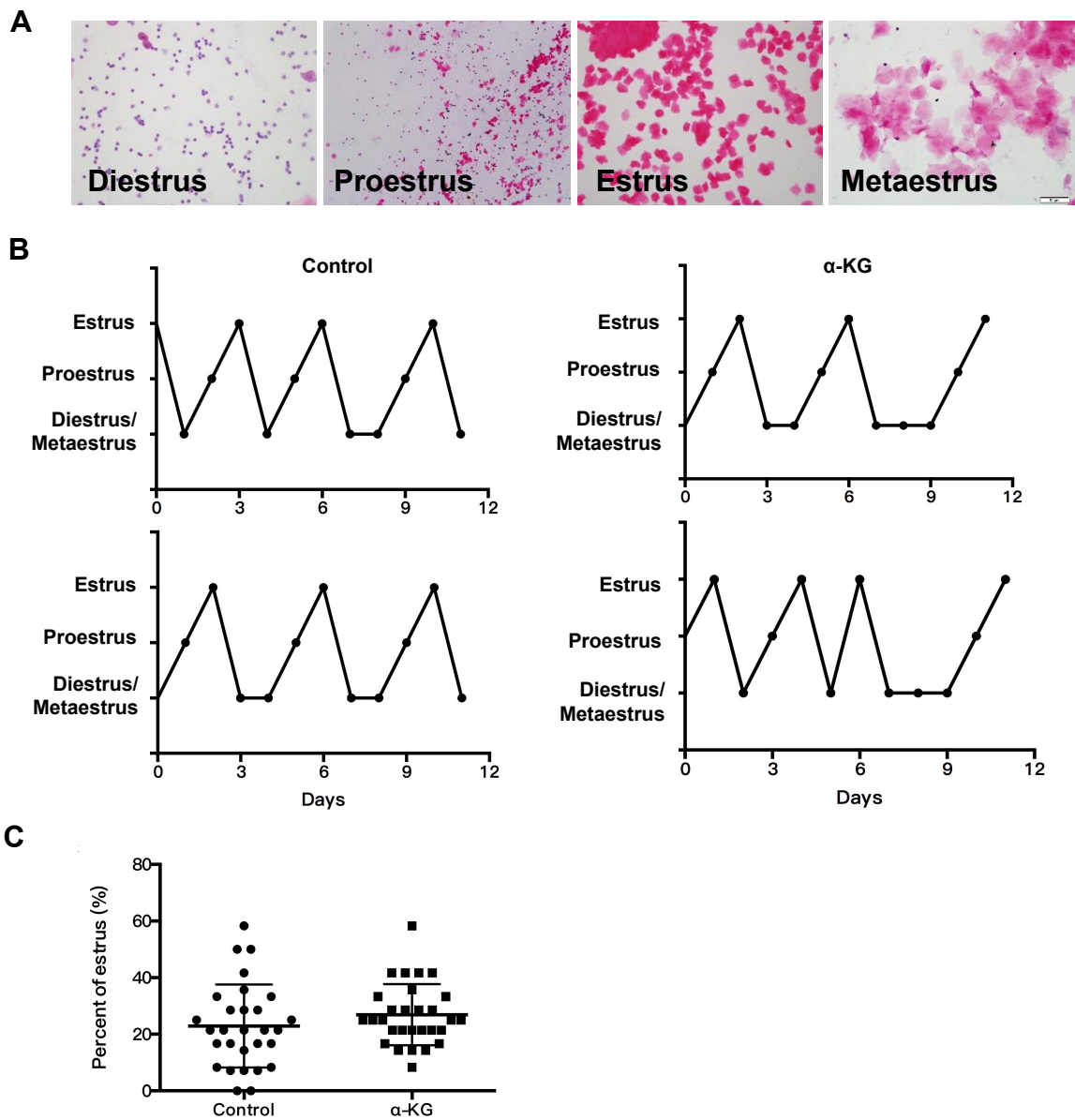

Supplement: Supplementary file 1 — Figure S1 [file ACEL-20-e13291-s001.pdf]

**S\_Figure 2**

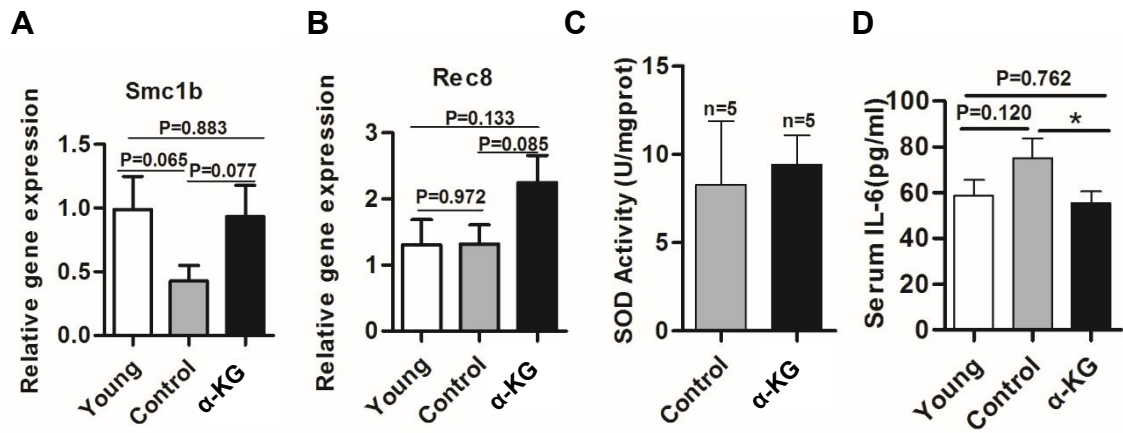

Supplement: Supplementary file 2 — Figure S2 [file ACEL-20-e13291-s002.pdf]

S\_Figure 3

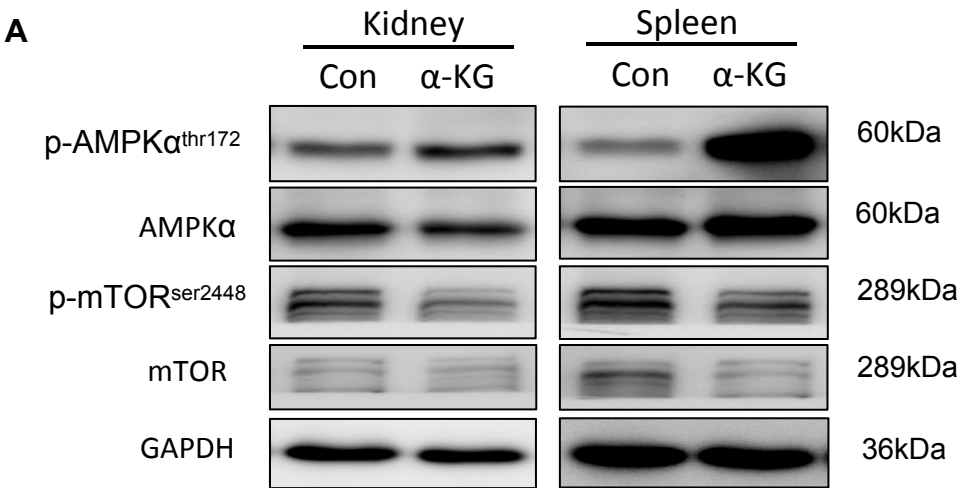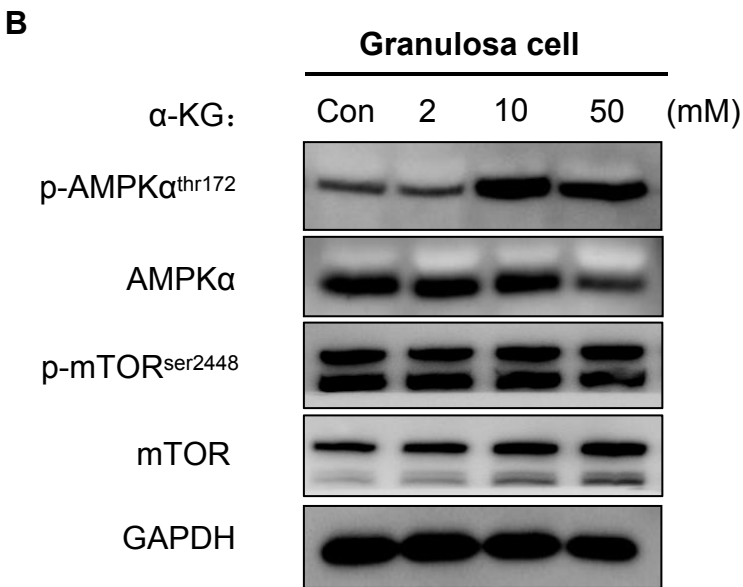

Supplement: Supplementary file 3 — Figure S3 [file ACEL-20-e13291-s003.pdf]

S\_Figure 4

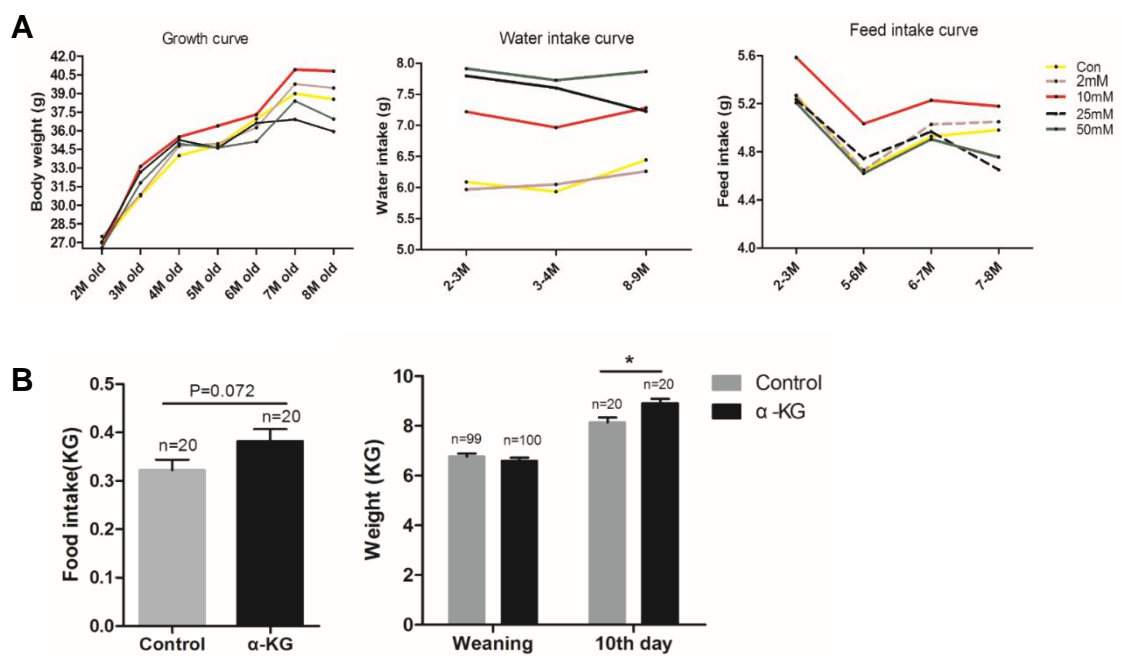

Supplement: Supplementary file 4 — Figure S4 [file ACEL-20-e13291-s004.pdf]

S\_Figure 5

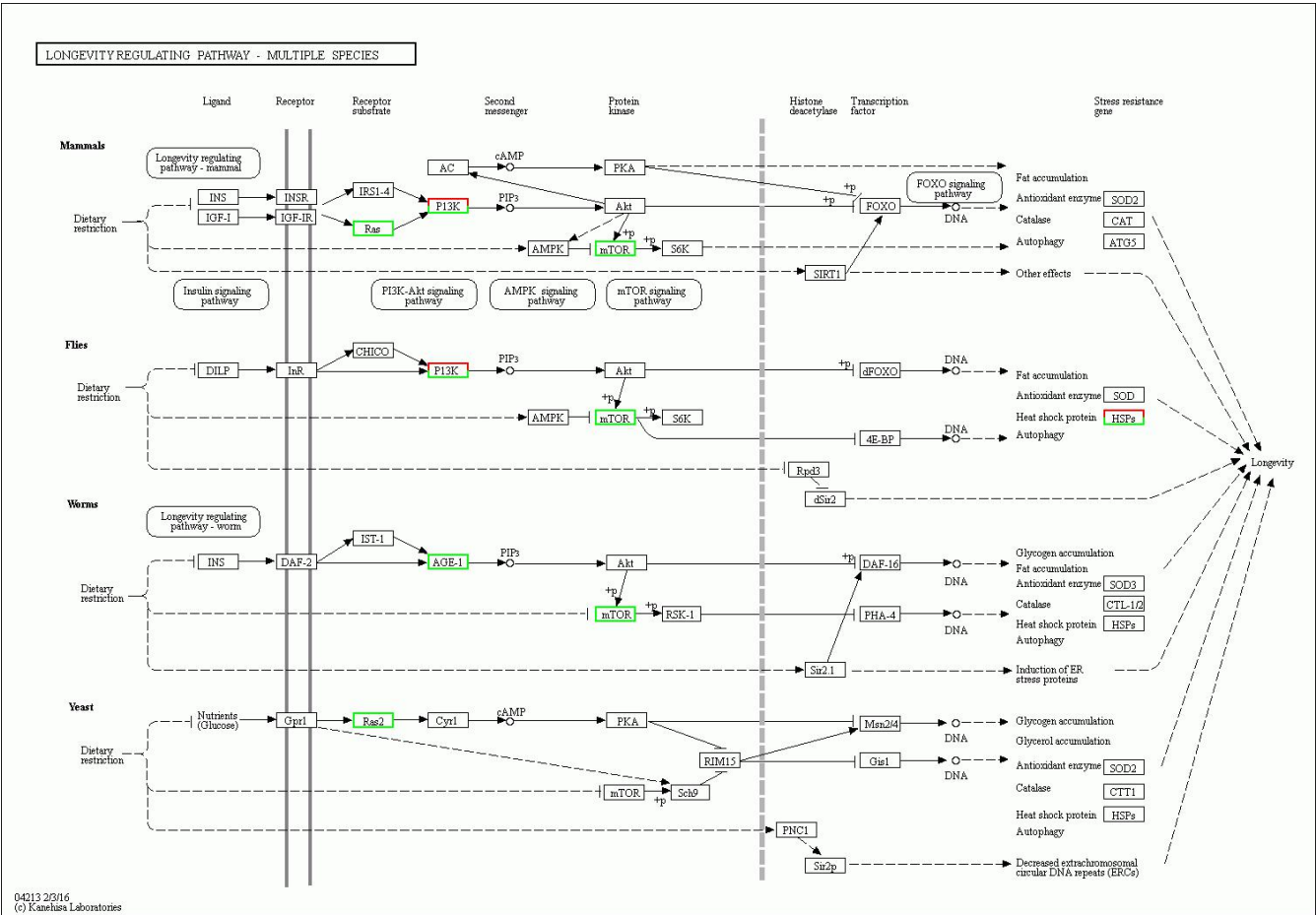

Supplement: Supplementary file 5 — Figure S5 [file ACEL-20-e13291-s005.pdf]
